# Recent Development in Micromanufacturing of Metallic Materials

**DOI:** 10.3390/ma13184046

**Published:** 2020-09-11

**Authors:** Jingwei Zhao, Zhengyi Jiang, Leszek A. Dobrzański, Chong Soo Lee, Fuxiao Yu

**Affiliations:** 1 College of Mechanical and Vehicle Engineering, Taiyuan University of Technology, Taiyuan 030024, China; jzhao@tyut.edu.cn; 2 School of Mechanical, Materials, Mechatronic and Biomedical Engineering, University of Wollongong, Wollongong, NSW 2522, Australia; 3 Medical and Dental Engineering Center for Research, Design and Production ASKLEPIOS, ul. Krolowej Bony 13D, 44-100 Gliwice, Poland; 4 Graduate Institute of Ferrous Technology, Pohang University of Science and Technology (POSTECH), Pohang 37673, Korea; cslee@postech.ac.kr; 5 School of Materials Science and Engineering, Northeastern University, Shenyang 110819, China; fxyu@mail.neu.edu.cn

**Keywords:** micromanufacturing, metallic materials, miniaturization, micro products

## Abstract

Product miniaturization is a trend for facilitating product usage, enabling product functions to be implemented in microscale geometries, and aimed at reducing product weight, volume, cost and pollution. Driven by ongoing miniaturization in diverse areas including medical devices, precision equipment, communication devices, micro-electromechanical systems (MEMS) and microsystems technology (MST), the demands for micro metallic products have increased tremendously. Such a trend requires development of advanced micromanufacturing technology of metallic materials for producing high-quality micro metallic products that possess excellent dimensional tolerances, required mechanical properties and improved surface quality. Micromanufacturing differs from conventional manufacturing technology in terms of materials, processes, tools, and machines and equipment, due to the miniaturization nature of the whole micromanufacturing system, which challenges the rapid development of micromanufacturing technology. Against such a background, the Special Issue “Micromanufacturing of Metallic Materials” was proposed to present the recent developments of micromanufacturing technologies of metallic materials. The papers collected in the Special Issue include research articles, literature review and technical notes, which have been highlighted in this editorial.

Quo Vadis, Munde? (in Latin—Where are you going world?) (It is a paraphrase of the words of the Apostle Peter “Quo Vadis, Domine”, according to a legend fleeing Rome in fear of persecution and death, directed to Jesus Christ going in the opposite direction without fear. “Quo Vadis” is the title of the famous novel of the Polish writer Henryk Sienkiewicz, who received the Nobel Prize in Literature in 1905.). It seems to be one of the most critical questions that millions and maybe even billions of people are asking themselves. Just a few months ago, despite the areas of poverty and local wars, the answer was obvious. The idea was to make living conditions as pleasant as possible and to continuously improve them, and to do so to affect more and more of the world’s citizens. The UN has defined these perspectives by formulating seventeen Sustainable Development Goals [1]. However, it turned out that the tiny creature of nature, the severe acute respiratory syndrome coronavirus 2 (SARS-CoV-2) with a diameter 100 trillion times smaller than the diameter of the Earth can completely block the course of world events and lead to its lockdown. This virus just caused the coronavirus disease (COVID-19) and the associated global pandemic. It, of course, will leave its mark on the coming years, maybe even decades.

Some development trends, however, seem to be still valid after a short stop. The Japanese government addressed the problem most widely when formulating the Society 5.0 program [2,3,4,5,6,7]. It concerns the development of civilization ranging from hunting and agriculture in primitive times to the developed IT society today. Previously created in Germany [8,9,10] and adopted in the European Union [11] and in many other countries, the program covers the next stages of the technological revolution. The current stage of Industry 4.0 marks the beginning of an era of digital industrial technology in which, in addition to people supporting automated production, systems, sensors, machines, metadata and IT, including the internet of people, things and services, which connects various participants in the value chain in the production process, through a network internally and inter-organizationally [12,13,14,15]. In the technological aspect, a key role is played by cyber-physical systems (CPS), similar to other cyber-social, cyber-business, and cyber-biological systems covering numerous algorithms, communication infrastructure, and high-computing cyberspace for developing and processing metadata. In industrial organizations, it results in improved security, operational efficiency, and data collection efficiency, as well as significant savings. CPS systems interact with each other and with neighboring smart components, communicating between machines and smart products. Smart production takes place in smart factories constituting production systems, where people, machines and sensors interact in real-time, and production decisions are made based on experiments and simulations of the real conditions of manufacturing products made in virtual reality. A virtual copy of the physical world in the form of a digital twin enables computer simulations and prediction, which is aided by metadata sets and self-education systems and the use of artificial intelligence methods. The nine technologies determining progress in Industry 4.0 include big data sets, autonomous robots, simulations, integration of horizontal and vertical systems, the internet of things, cybersecurity, cloud computing, additive manufacturing and augmented reality [16,17]. The model presented in this way, however, seems to be only a fragment of reality and is, therefore, a simplification. The full augmented holistic model of Industry 4.0 is pictured by a regular octahedron containing people as the causative agent, a technological platform with four complementary components, and smart products as the goal of the whole activity [18,19,20]. At the technological platform, the development of engineering materials and product manufacturing processes are taken into account, including additive technologies, development of technological machines, and CPS systems, which are in fact only one of the six components of the full augmented holistic model of Industry 4.0.

This approach to the problem clearly indicates the contemporary importance of both material and technological design [12,13,14,15,18,19,20,21]. However, it is not only additive technologies that determine technological success. Among modern products enabling universally expected improvement in the quality of life, health, and work efficiency are ever newer models of mobile phones and smartphones, CD and MP3 players, iPods, wide flat-screen displays and computers, including those installed in many market products, including for automotive, aviation and space applications, as well as in systems and manufacturing machines. The devices include pressure, thermal, temperature, gas, mass flow, speed, and sound sensors, injection nozzles, electrical, pneumatic and thermal micro-actuators, chemical micro-reactors, micro-motors, micro-gears, micro-valves, micro-fans, micro-tools, micro-molding, and micro-replication molds. These devices are examples of the now more and more miniaturized products, systems and devices, which also include, for example, micro-systems, including micro- and nano-electromechanical (MEMS and NEMS), micro-mechanical, micro-medical and micro-optical electronic-mechanical (MOES) systems, microreactors, fuel cells, and numerous micro-devices and sensors widely used among other applications, in automotive, aviation industries, telecommunications, and IT facilities. It is worth noting that the modern manufacturing of many of the above-mentioned micro- and even nano-elements requires compliance with the rules of the Industry 4.0 stage of the technological revolution, and on the other hand, their production is a prerequisite for the implementation of this modern Industry 4.0 technology, where different sensors and devices automatically identify raw materials, semi-finished products, and ready-made components and products. Micro-components are often used in various clinical areas of medicine and dentistry, including on cardiovascular sensors, microprocessor ceramic packaging, implantable devices, medical instruments, and implants, as well as coatings on micro polymers or metal implant components [22,23]. Currently, there is a significant variety of micro-products.

Nanomaterials, coatings and thin films coating the working surfaces of many products and components are becoming increasingly important, including for their use in micro- and nano-devices, sensors, in communication, medicine, and dentistry, but also in household appliances, sound and television sets, watches and many other products used daily by millions of people. Properly designed and manufactured material is often a determinant of technological progress and a prerequisite for the implementation of the design intention for micro-components and micro-devices. They play important roles in material processing and plastic deformation at the micro- and nano-scale, where their characteristics and properties include the structure of materials and nanomaterials, their phase composition, chemical purity, grain size, grain boundary structure, phase transformation micromechanisms, precipitation processes, dispersion and size of precipitates and intermetallic phases, plastic deformation micromechanisms, and surface layer deposition processes. The mechanical and physicochemical properties of these materials, as well as their mechanisms to resist material damage are also important for prevention of corrosion, abrasion, fatigue, the influence of elevated temperature, and even creep. These factors are important for determining the maintenance of micro-elements and micro-devices and require precise design before starting production and require consideration during their exploitation.

Micromanufacturing, together with nanotechnology, has now become a common technological practice [24,25,26,27,28]. Therefore, it is necessary to continually increase the knowledge of scientists, engineers, and managers involved in this production. Micromanufacturing includes production methods, technologies and systems that meet the harsh rigors of Industry 4.0, as well as development of suitable materials, machinery, tools, and organizational strategies and production management methods. The specificity of this production, mainly due to the miniaturized scale of products, requires separate design and manufacturing experience, the use of specific materials and adequate manufacturing equipment, which are often also miniaturized, and requires special care for the mechanization, automation, and computerization of the technologies used, and therefore this field is often combined and referred to as microsystems technology (MST). Among the materials used can be specified silicon, considered to be “technologically mature” and currently more and more often used rather than other engineering materials; materials used are very usually metal, but also composites, as well as polymers and ceramics. Generally, two main groups can be considered among MST products. MEMS can be regarded as a classic application; its uses include photolithography, plating, laser ablation, chemical etching, hybridized lithography and electroplating and molding known as LIGA (a German acronym for **Li**thographie, **G**alvanoformung, **A**bformung). The other group consists of other manufactured micro-products, not included in MEMS, using micro-mechanical cutting, laser cutting/drilling/patterning, micro-injection, micro-extrusion, micro-embossing, micro-molding, micro-stamping, as well as electrical discharge machining (EDM), also known as wire erosion, wire burning, die sinking, spark eroding or spark machining. This group of new processes for the production of micro products/features or so-called micro-machining also uses conventional technologies with a correspondingly reduced scale, which includes micro-machining (mechanical, thermal, electro-chemical, electrical discharge), micro-molding/replication, micro-formation methods (quick methods, electro molding, injection molding) and micro-joining.

Another goal of micromanufacturing is the production of products/devices using miniaturized machines/systems, usually instead of universal large-sized machines/systems. The production of micro-products and associated micro-production strategies differ from those used on a conventional scale. The individual components/products are manufactured at one or several manufacturing stations, employing subtractive machining or plastic deformation, as well as additive methods, to finally assemble or connect them by welding methods and/or joining materials. In the case of micro-products, deposition, layering, and/or pattering methods are used, with technological operations most often performed using one machine or technology platform in combination with integrated packaging and/or assembly operations. Conventional methods as well as hybrid production combining all or some of the subtractive, additive, forming, and/or joining technologies can also be used. Due to the use of various energy sources, the technologies used to produce micro-components/products can be mechanical, electrical, electro-chemical, chemical, laser, or electronic. As a rule, dedicated devices are used, enabling the implementation of a predetermined technological process, although more and more interest of producers of micro-products, and more importantly, manufacturers of technological machines, employ multi-process devices whose working platform allows a hybrid combination of various manufacturing processes, e.g., micro-milling, turning, grinding, buffing, polishing, micro-electro discharge machining, micro-electrochemical machining and/or laser machining, without having to clamp the workpiece/product each time. Favorable solutions in this area bode very well for the development of integrated micro-machining technologies.

The attractiveness of this avant-garde technology for micromanufacturing of metallic materials for the production of micro-components/products has prompted and encouraged us to prepare a Special Issue on “Micromanufacturing of Metallic Materials”. Miniaturization of products is a trend that facilitates the use of the product, enabling the implementation of product functions in microscale geometry, aimed at reducing mass, volume, costs, and environmental pollution through the manufacture of this micro-product. From around 30 papers submitted, after careful selection as a result of the opinion-giving process, 14 papers were selected and published in this book. With the exception of one literature review and one other publication, the others are research papers. These papers have been prioritized.

Carpenter and Tabei [29] conducted a literature review on residual stress development, prevention and compensation in metal additive manufacturing, in which a holistic calculation framework was proposed to control the level of residual stresses by optimizing the conditions of the additive manufacturing processes. Also, various additive manufacturing technologies and residual stress sources, residual stress measurement techniques, and their dependence on additive manufacturing (AM) process conditions were briefly described, and current modeling methods were proposed to prevent permanent deformation of manufactured products using additive manufacturing methods.

Máthis et al. [30] have prepared a paper on the micro-tensile behavior of Mg-Al-Zn alloy processed by equal channel angular pressing (ECAP). In this research, AZ31 magnesium alloy was extruded four times in an equal rectangular channel. Deformation characteristics (yield stress, ultimate tensile stress, and uniform elongation) exhibit significant anisotropy as a consequence of different orientations between the stress direction and texture, and thus different deformation mechanisms were confirmed on the samples obtained using the electron backscatter diffraction (EBSD) technique.

The paper on bio-inspired functional surface fabricated by electrically assisted micro-embossing of AZ31 magnesium alloy was developed by Wang et al. [31]. This research focuses on the use of different current densities to perform embossed micro-channels in textured bulk metallic glass dies. These dies are prepared by thermoplastic forming based on the compression of photolithographic silicon molds. The results show that large areas of bio-inspired textures could be fabricated on magnesium alloy. Filling depth and depth–width ratio nonlinearly increases when higher current densities are used, and the temperature is kept below the temperature of the glass transition to avoid melting and to avoid an early breakage of the die. Electrically assisted micro-forming has demonstrated the ability to reduce size effects, improving formability and decreasing flow stress, making it a promising hybrid process to control the filling quality of micro-scale features.

Thangaraj et al. [32] conducted research to enhance the surface quality of a micro titanium alloy specimen in a wire electrical discharge machining (WEDM) process by adopting Taguchi–Grey relation analysis (TGRA)-based optimization. The surface measures of machined titanium alloys as dental materials can be enhanced by adopting a decision-making algorithm in the machining process. In the present study, Taguchi–Grey analysis-based criteria decision making was applied to the input process factors in the wire electric discharge machining (EDM) process. It was proved that the proposed method can enhance the efficacy of the process.

The team of Xue et al. [33] prepared a paper on an electrically-assisted rolling process of corrugated surface microstructure with T2 copper foil. Electrically-assisted forming is a low-cost and high-efficiency method to enhance the formability of materials. The electric current reduces the flow stress and the fracture strain. The study proves that the current can improve the forming quality of the corrugated foils and is a promising surface texture forming process.

The paper on adaptive spiral tool path generation for diamond turning of large aperture freeform optics was developed by Wang et al. [34]. This paper reports a novel adaptive tool path generation for slow tool servo slow tool servo (STS) iamond turning. Comparison of the surface generation of typical freeform surfaces with adaptive tool path generation and the commercial software DiffSys is conducted both theoretically and experimentally. The adaptive tool path generation can effectively reduce the volume of control points, decrease the vibration of side-feeding motion, and improve machining efficiency while surface quality is well maintained for large aperture freeform optics.

Liu et al. [35] conducted an experimental investigation on form error for slow tool servo diamond turning of micro lens arrays on a roller mold. In this study, a novel forming approach based on a slow tool servo is presented to fabricate microlens arrays on an aluminum alloy (6061) roller mold. Based on the different distribution patterns of the discrete points of the microlens, the equal-arc method and the equal-angle method are also proposed to generate the tool path. According to the kinematic analysis of the cutting axis, the chatter mark results from the overlarge instantaneous acceleration oscillations of the cutting axis during the slow tool servo diamond turning process of the microlens arrays. The results are acquired with fine surface quality. Slow tool servo assisted ultra-precision diamond turning is a promising machining process with high accuracy and low cost to generate the large-area microlens arrays on a roller mold.

The paper on microsheet metal deformation behaviors in ultrasonic-vibration-assisted uniaxial tension with aluminum alloy 5052 was prepared by Wang et al. [36]. In this investigation, ultrasonic-vibration-assisted uniaxial tensile experiments were carried out utilizing GB 5052 thin sheets of different thicknesses and grain sizes, respectively. The uniform deformation ability of thin sheets could be improved by increasing the hardening exponent with ultrasonic-vibration. The authors found that ultrasonic-vibration is helpful in improving the forming limit in micro sheet forming, e.g., microbulging and deep drawing processes.

Supercooled Zr35Ti30Cu8.25Be26.75 metallic glass is the topic of research in the paper entitled “Ultrasonic Vibration Facilitates the Micro-Formability of a Zr-Based Metallic Glass”, which was written by Han et al. [37]. Ultrasonic vibration was introduced as an effective method to improve the micro-formability of metallic glasses, owing to its capabilities of improving the material flow and reducing interfacial friction. The more intriguing finding is that the micro formability of the Zr-based metallic glasses can be further improved by tuning the amplitude of the ultrasonic vibration. The results were demonstrated by the finite element method.

Ren et al. [38] conducted a numerical research on slip system evolution in ultra-thin stainless steel foil, in which a three-dimensional uniaxial tension model was established, based on the crystal plasticity finite element method and Voronoi polyhedron theory. The number and characteristics of active slip systems and the deformation degree of the grain are different due to the different initial grain orientations. The slip systems preferentially initiate at grain boundaries and cause slip system activity at the interior and free surface of the grain. The brass, S, and copper oriented 304 stainless steel foil exhibit a high strain hardening index, which is beneficial to strengthening. However, the cube and Goss oriented 304 stainless steel foil has a low deformation resistance and is prone to plastic deformation.

Suzuki et al. [39] elucidated the shearing mechanism of finish-type FB and extrusion-type FB for a thin foil of JIS SUS304 by numerical and EBSD analyses. In this research, a numerical analysis using finite element (FE) analysis was performed to clarify the shearing mechanism in the process of extrusion-type fine blanking (FB) for a thin foil of JIS SUS304. The principal stress near the shearing surface has mostly compressive components in extrusion-type FB due to its negative clearance, and the critical fracture value was also less than that in the finish-type FB, in which the principal stress near the shearing surface has mostly tensile components. Using SEM observations with electron backscatter diffraction (EBSD) analysis of the shearing surface, it was confirmed that the reductions in deformation-induced crystal orientation rotation and martensite transformation in extrusion-type FB are present in comparison with those in finish-type FB. Suzuki et al. [40] also prepared an article titled “Optimum Clearance in the Microblanking of Thin Foil of Austenitic Stainless Steel JIS SUS304 Studied from Shear Cut Surface and Punch Load”. An extrusion-type fine blanking with a negative clearance was proposed by the authors instead of standard fine blanking, for creating a full-sheared surface in the micro blanking process. It was clarified that the clearance at which the cut surface does not fracture and minimization of the punch load is achieved is gained by the use of clearance—4 μm.

The paper on the fabrication of a micro-punch array by plasma printing for micro-embossing into copper substrates was prepared by Shiratori et al. [41]. Copper substrates were wrought to have micro-grooves for packaging by micro-stamping with the use of an AISI316 stainless steel micro-punch array. A negative pattern was printed directly onto the AISI316 die substrate, which was plasma nitrided. The printed surfaces were selectively sand-blasted to fabricate the micro-textured punch array for micro-embossing. Since the nitrogen supersaturated heads had sufficient hardness against the blasting media, the printed parts of AISI316 die were removed.

Machno et al. [42] contributed a paper on the subject “Impact of the Deionized Water on Making High Aspect Ratio Holes in the Inconel 718 Alloy with the Use of Electrical Discharge Drilling”. This paper includes an analysis of the influence of the machining parameters (pulse time, current amplitude and discharge voltage) on the process performance (drilling speed, linear tool wear, taper angle, the hole’s aspect ratio, and side gap thickness), during electrical discharge drilling (EDD) with the use of deionized water in the Inconel 718 alloy. An analysis of the results indicates increasing of the hole’s aspect ratio by about 15% (above 30), decreasing the side gap thickness by about 40%, and enhanced surface integrity.

In summary, it is difficult to expect that in a scholarly book with a relatively limited volume, all issues raised by a modern approach to micromanufacturing could be included. This is simply impossible because of the very wide scope of this super modern technological approach. Therefore, it was only possible to give reasonably representative examples, and that is how it happened. We offer readers very interesting, in our opinion, research material. If at least one of the presented papers turns out to be interesting for the reader who reached for this book, we will have the basis to believe that as editors, we have achieved our goals. It remains for us to wish you fruitful reading.
